# Exploring the intricate relationship between miRNA dysregulation and breast cancer development: insights into the impact of environmental chemicals

**DOI:** 10.3389/fimmu.2024.1333563

**Published:** 2024-05-14

**Authors:** Narges Abolhasanzadeh, Sajed Sarabandi, Bahar Dehghan, Vahidreza Karamad, Cigir Biray Avci, Behrouz Shademan, Alireza Nourazarian

**Affiliations:** ^1^ Department of Biology, Faculty of Natural Sciences, University of Tabriz, Tabriz, Iran; ^2^ Department of Computer Science Leiden University, Leiden, Netherlands; ^3^ Department of Medical Biology, Ege University Medical School, Izmir, Türkiye; ^4^ Stem Cell Research Center, Tabriz University of Medical Sciences, Tabriz, Iran; ^5^ Department of Basic Medical Sciences, Khoy University of Medical Sciences, Khoy, Iran

**Keywords:** breast cancer, microRNAs, environmental chemicals, heavy metals, air pollutants

## Abstract

Breast cancer stands as the most prevalent form of cancer among women globally, influenced by a combination of genetic and environmental factors. Recent studies have investigated changes in microRNAs (miRNAs) during breast cancer progression and the potential impact of environmental chemicals on miRNA expression. This review aims to provide an updated overview of miRNA alterations in breast cancer and to explore their potential association with environmental chemicals. We will discuss the current knowledge on dysregulated miRNAs in breast cancer, including both upregulated and downregulated miRNAs. Additionally, we will review the influence of environmental chemicals, such as endocrine-disrupting compounds, heavy metals, and air pollutants, on miRNA expression and their potential contribution to breast cancer development. This review aims to advance our understanding of the complex molecular mechanisms underlying miRNA dysregulation in breast cancer by comprehensively examining miRNA alterations and their association with environmental chemicals. This knowledge is crucial for the development of targeted therapies and preventive measures. Furthermore, identifying specific miRNAs affected by environmental chemicals may allow the prediction of individual susceptibility to breast cancer and the design of personalized intervention strategies.

## 1 Introduction

Breast cancer stands as the most prevalent cancer among women, contributing to 6.9% of all cancer-related fatalities, positioning it as the fifth-leading cause of cancer-related death. Notably, breast cancer mortality rates are significantly higher in developing countries than in developed countries (1). Several lifestyle and reproductive factors are associated with this type of cancer: early onset of menstruation, delayed onset of menopause, postponed childbirth, abbreviated breastfeeding periods, postmenopausal hormone replacement therapy, usage of oral contraceptives, alcohol consumption, and obesity (2). Hereditary mutations in certain genes, particularly BRCA1 and BRCA2, are associated with approximately 5%–10% of breast tumors (3). Gene expression profiling techniques have revealed numerous gene dysregulations in breast cancer samples, further demonstrating the complex genomic nature of breast cancer (4).

MicroRNAs (miRNAs) are short strands of RNA, typically 20–24 nucleotides in length, originating from DNA transcription but not translated into proteins. These molecules are involved in the post-transcriptional repression of the expression of specific genes by binding to the 3’ untranslated regions of target messenger RNAs (mRNAs) (5). miRNAs play a crucial role in governing the regulation of both transcriptional and post-transcriptional gene expression by engaging in specific interactions with target mRNAs (6). Recent studies have reported the dysregulation of microRNAs (miRNAs) in both tissue and plasma samples from breast cancer patients and correlated these findings with elements of tumorigenesis (7–9). Observations have reported alterations in miRNA expression in breast cancer samples, with instances such as abnormal upregulation of miR-221 and miR-222 in breast cancer occurrences (10, 11).

Individuals are constantly exposed to environmental chemicals in their daily lives. These chemicals are found in items such as plastic food and beverage containers, cosmetics, sunscreens, and cleaning products. In addition, chemical pesticides are often found as residues on commercially grown crops such as fruits, vegetables, and grains. Widespread exposure to these environmental chemicals is a known contributor to the increased risk of several diseases (12, 13). The classification of these chemicals, based on their ability to alter DNA sequence, is of paramount importance in understanding their causal relevance. These data are essential for assessing environmental hazards and guiding existing regulatory measures to protect against exposure (14). However, genetic mutations have been shown to contribute to only a small fraction of environmentally-induced diseases (15). MiRNAs have recently been recognized as a contributing element in gene expression, potentially linking environmental chemicals to associated diseases. Research has indicated an increase in toxic metals such as iron, copper, zinc, lead, chromium, and nickel in malignant breast tumors, as documented by advanced analytical methods such as atomic absorption spectrophotometry and energy-dispersive spectrometry. Elevated metal concentrations have been associated with adverse molecular prognostic indicators (16). Exposure to toxic metals induced tumorigenesis and led to increased expression of human epidermal growth factor receptor 2 (HER2/neu), p53, Ki-67, and O6-methylguanine DNA methyltransferase, while suppressing estrogen receptor-alpha (ER-α) and progesterone receptor (PR) expression. At the same time, researchers have linked pathological DNA methylation to the accumulation of toxic metals in tumor tissue. Both malignant and benign breast tumors had higher concentrations of trace elements such as calcium, iron, copper, and zinc compared to normal tissues (17). Significant statistical associations were found between trace elements and accepted breast cancer prognostic factors. Interestingly, copper showed a significant association with overall survival, highlighting its potential as a biomarker. Furthermore, prolonged exposure to cadmium was found to induce migration and invasion of breast cancer cells via the TGIF/MMP2 signaling pathway (18). Tungsten was found to specifically affect the tumor microenvironment, primarily leading to increased metastasis in breast cancer (19).

The review thoroughly evaluates a large number of studies and provides an up-to-date and detailed summary of the most recent research findings. The authors highlight the influence of environmental chemicals on miRNA expression and how these changes may contribute to breast cancer development. The paper also details the challenges in the field and provides future directions to inspire ongoing research and exploration.

## 2 Alterations of miRNA in breast cancer

miRNAs are endogenous regulators of gene expression that are synthesized in the body and have the ability to modulate gene expression. These RNA molecules are single-stranded and non-coding, typically consisting of approximately 24 nucleotides in their active state (20). Notably, the expression of miRNAs varies depending on the tissue, a difference observed in both normal and cancer cells. This highlights the central role of miRNAs in pathological processes (21). Several miRNAs act as either tumor suppressors or oncogenes by targeting genes associated with cancer. Between 30% and 80% of protein-coding genes have at least one binding site for miRNA, giving miRNAs the ability to control the expression of multiple mRNAs by partially binding to them (22). MiRNAs are critical for the regulation of gene expression because they specifically target mRNA and implement various post-transcriptional mechanisms, such as translation inhibition and mRNA degradation (23). The efficacy of this regulatory mechanism largely depends on the degree of complementarity between miRNA and mRNA. High complementarity can result in damage to the target mRNA, while low complementarity triggers the process of translational repression (24). As a result of these variations, changes in miRNA expression have been observed in breast cancer (**Table 1**).

**Table 1:** Some studies that investigated the effect of miRNAs on the biological characteristics of breast cancer cells by targeting specific genes.

Study	Samples	Upregulated miRNA	Downregulated miRNA	Target gene	Result	Ref.
Long et al., 2020	MCF-7, MDA-MB-231 cell lines	–	miR-99a	FGFR3	The miR-99a/FGFR3 axis plays a crucial role as a tumor regulator in breast cancer.	(25)
Zhang et al., 2020	MCF-7, MDA-MB-231 cell lines	miR-21-5p	–	SPRY2	The synergistic impact of combining the miR-21-5p inhibitor with reversine was observed through the regulation of SPRY2.	(26)
Wang et al., 2019	Plasma	miR-21	–	LZTFL1	LZTFL1 is targeted by miR-21, leading to the promotion of breast cancer proliferation and metastasis.	(27)
Jansson et al., 2014	MCF7, HEK293/BJ-hTERT cells	–	miR-339-5p	MDM2	Disrupting the function of miR-339-5p hinders the p53 response in cancer cells, leading to an elevated rate of proliferation.	(28)
Pan et al., 2009	MCF-7, MCF-7-MX100 cells	miR-328	–	BCRP/ABCG2	miR-328 regulates the expression of the ABCG2 protein by targeting its ABCG2 3’-UTR, thereby influencing drug disposition in human breast cancer cells.	(29)
Yang et al., 2023	Breast cancer tissues/BT549 and MCF-7 cells	–	miR-874-3p	VDAC1	By targeting VDAC1, miRNA-874-3p suppresses the migration, invasion, and proliferation of breast cancer cells.	(30)
Mohammaddoust et al., 2023	MCF-10, MCF-7/MDA-MB-231 cell lines	miR-183	–	PTEN	The reduction of PTEN expression by miR-183 may be a crucial factor in the advancement of breast cancer.	(31)
Shi et al., 2024	Breast cancer tissues/SKBR3, MDA-MB-231, and MCF7	miR-103a-3p	–	ETNK1	The inhibition of ETNK1 by miR-103a-3p may facilitate the progression of breast cancer.	(32)
Zhao et al., 2024	Breast cancer cells	–	miR-600	EZH2/RUNX3	miR-600 has the capability to impede the malignant behavior of breast cancer cells and enhance their sensitivity to sorafenib through the EZH2/RUNX3 axis.	(33)
Patel et al., 2016	MCF-7, MDAMB-231 cells	–	miR-15a, miR-16	BMI1/BCL2	BMI1 is implicated in the induction of apoptosis in breast cancer cells by miR-15a/miR-16.	(34)
Shi et al., 2015	Primary breast cancer tissues/BT549, MDA-MB-231, MDA-MB-468, MCF7, SK-BR-3, T47D cells	miR-7-5p	–	REGγ	By targeting REGγ, miR-7-5p inhibits cell proliferation and triggers apoptosis.	(35)
Chen et al., 2017	MCF-7, T47D, SKBR3, BT549, MDA-MB-231, MDA-MB-435S cells	miR-23a	–	XIAP	*In vitro*, the enforced expression of miR-23a downregulated XIAP expression, thereby promoting autophagy, colony formation, migration, and invasion of breast cancer cells.	(36)
Xia et al., 2017	Adjacent normal breast tissues/MCF-7 cells	miR-32	–	FBXW7	By targeting FBXW7, miR-32 promotes cell proliferation and migration while suppressing apoptosis.	(37)
Gu et al., 2020	T47D, MCF-7, ZR-75-1 and HCC1954 cells	–	miR-139-5p	COL11A1	Either silencing COL11A1 or overexpressing miR-139-5p can both inhibit proliferation and promote apoptosis.	(38)

Leucine zipper transcription factor-like 1 (LZTFL1); silent information regulator 1 (SIRT1); breast cancer resistance protein (BCRP/ABCG2); voltage-dependent anion channel 1 (VDAC1); proteasome activator subunit 3 (REGγ); X-linked inhibitor of apoptosis (XIAP); collagen type XI alpha 1 chain (COL11A1).

Breast cancer, excluding skin cancer, is the most frequent cancer diagnosis for women globally. The statistics show that approximately 13% of women worldwide will receive a diagnosis of invasive breast cancer (39). Breast cancer is the second leading cause of death for women, just behind lung cancer (40, 41). The classification of breast cancer is found on molecular and histological subtypes. The molecular classification of breast cancer takes into account hormone receptors (estrogen and progesterone) and the presence or absence of the HER2-positive/HER2-negative protein. Histologically, breast cancer is classified as invasive ductal carcinoma, invasive lobular carcinoma, or mixed ductal/lobular carcinoma. The most common subtype is HR-positive/HER2-negative invasive ductal carcinoma (40, 42).

**Figure 1:**
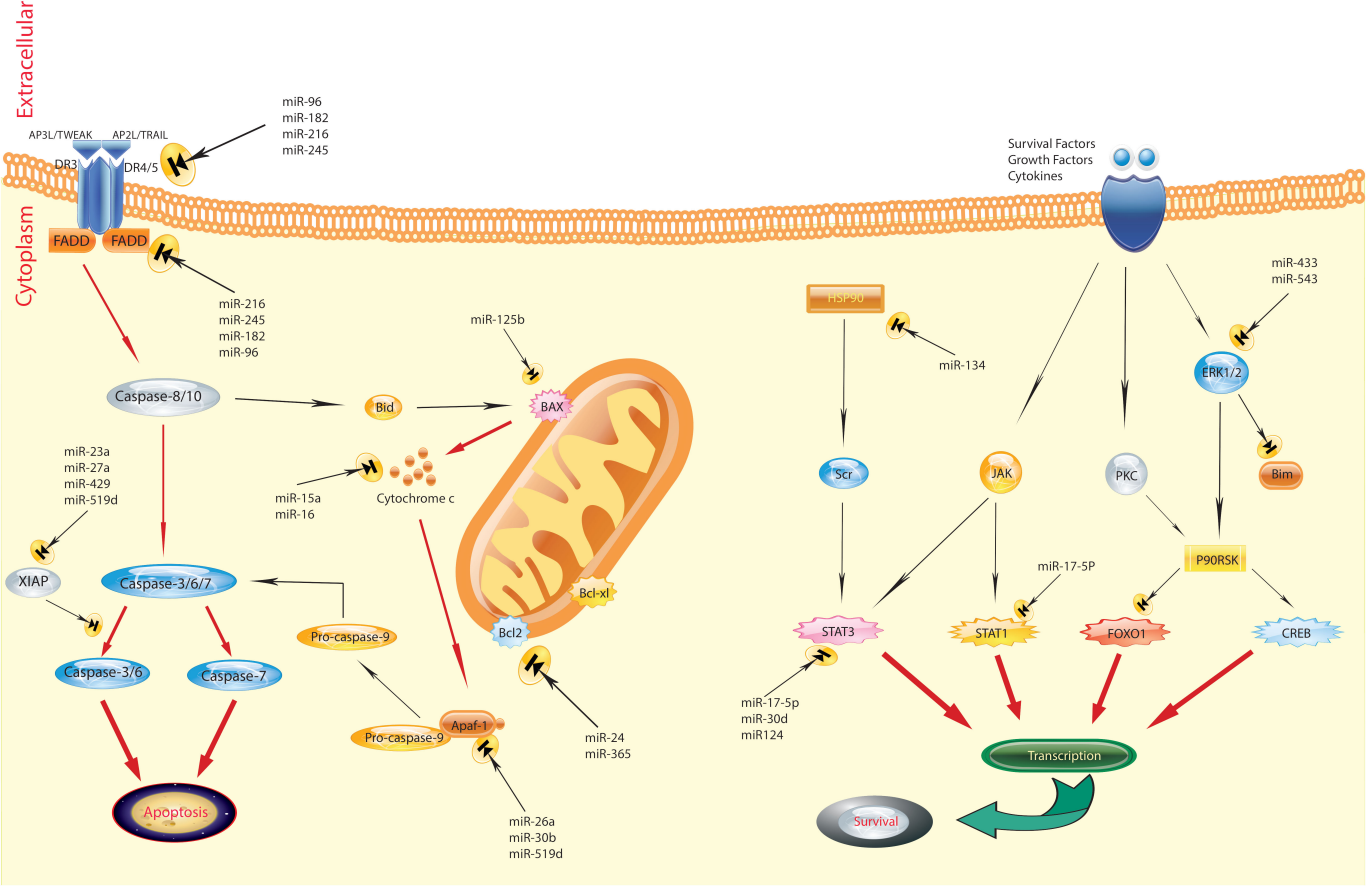
Regulation of apoptosis by miRNAs in breast cancer. The figure shows that the aberrant expression of various miRNAs could contribute to adversely modulating the mitochondrial pathway of apoptosis, which is involved in triggering human breast cancer. Several pathways are involved in the regulation of apoptosis. Defects can happen at any portion of these pathways, resulting in the malignant transformation of cells, facilitation of tumor metastasis, and induction of resistance to anticancer agents (43).

In recent years, miRNA has emerged as a significant biomarker in the progression of breast cancer, playing a crucial role in carcinogenesis (**Figure 1**) (43, 44). Zhao and colleagues (45) performed a study indicating that the levels of miR-372, a regulator of E2F1, were significantly decreased in tissue samples from patients with primary breast cancer, including both tumor and adjacent non-tumor tissues, as well as in several breast cancer cell lines (BT-474, MCF-7, MDA-MB-436, and MDA-MB-231), compared to normal breast tissue cell lines. This reduction in expression is attributed to its role in suppressing tumors in breast cancer development. This study also identified an upregulation of E2F1 in human breast cancer tissues compared to adjacent normal tissues. A notable negative correlation was observed between the mRNA levels of E2F1 and miR-372 in breast cancer tissues. These findings indicate that targeting E2F1 inhibits proliferation and induces apoptosis in breast cancer cells by miR-372 (45). However, Fan et al (46). present an alternative perspective, showing a significant increase in the expression of miR-372 in breast carcinoma tissues and cell lines (MCF-7, MDA-MB-231, and HCC38) compared to normal breast tissue and typical breast epithelial cells (MCF-10A). They argued that reducing the levels of miR-372 inhibits the proliferation of breast cancer cells. Their findings indicate that miR-372 functions as an oncogene in breast cancer cells, providing an alternative viewpoint on the role of miR-372 in breast cancer (46). Breast cancer prevalence and mortality rates are significant global concerns. Molecular and histological subtyping offer valuable insights into the nature of breast cancer. MiR-372, a type of miRNA, has been found to play both positive and negative roles in breast cancer development, underscoring the complexity of its involvement. Further research is required to fully comprehend the mechanisms and potential therapeutic implications of miR-372 in breast cancer.

In the breast cancer context, the miR-183/miR-96/miR-182 cluster present at the chromosome locus 7q32. 2 exhibits elevated expression levels across multiple cancer classifications. Zinc finger E-box binding homeobox 1 (ZEB1) and heat shock transcription factor 2 (HSF2) proteins contribute to the augmented transcription of this cluster (47). Additionally, activation of the hypoxia-inducible factor 1 (HIF-1) or PI3K/Akt pathway further enhances the miR-183/96/182 cluster (48). The increase in miR-96 promotes breast cancer cell growth and mobility by influencing the non-receptor type 9 gene of protein tyrosine phosphatase in MCF-7 breast cancer cells (49). Moreover, it is worth noting that the miR-183/96/182 cluster is present in exosomes obtained from patients with breast cancer, establishing its link to the disease. The miR-183/96/182 cluster serves as a cancer-promoting factor in the progression of breast cancer.

Additionally, high levels of miR-221/miR-222 expression led to the conversion of ER-α-positive tumors to ER-α-negative breast cancer by targeting the ER-α gene (50). Basal-like breast cancer exhibits enhanced transcription of miR-221/miR-222, which is driven by the basal-like transcription factor FOSL1 (also known as Fra1). This transcription factor facilitates the epithelial–mesenchymal transition (EMT) by interacting with the 3′ untranslated region (3′UTR) of trichorhinophalangeal 1 (TRPS1), resulting in heightened cellular migration and invasion capabilities (51). Furthermore, the miR-221/miR-222 cluster’s abnormal activity induces the self-perpetuation of breast cancer stem cells by targeting the phosphatase and tensin homolog (PTEN)-Akt signaling pathway (52). Therefore, it is recognized that miR-221/miR-222 functions as a noteworthy oncogene driving the advancement of aggressive ER-negative breast cancer.

In the context of breast cancer, the miR-199a/214 cluster on chromosome 1q24 generates three transcript types, namely, miR-199a-5p, miR-199a-3p, and miR-214. Multiple studies show that the hedgehog signaling pathway, the vitamin D receptor, and miR-214 exhibit involvement in a cross-talk axis that downregulates the expression of miR-199a/214 in breast cancer cells (53). The decrease in miR-199a/214 cluster expression results in a loss of its tumor-suppressive effects, specifically in triple-negative breast cancer (TNBC). Depletion of the miR-199a/214 cluster initiates an EMT-like phenotype in normal cell lineages (54). Conversely, elevating miR-199a/214 cluster expression has demonstrated the ability to hinder TNBC cell proliferation via inhibition of hedgehog signaling. This mechanism shows promise as a potential therapeutic approach in the treatment of breast cancer (55).

Additionally, the levels of enhancer of zeste homolog 2 (Ezh2) protein, a known factor promoting neoplasia in various tissues, have been linked to epigenetic silencing associated with the H3K27me3 modification. Researchers studying a breast cancer cell model have observed a significant inverse relationship between miR-199a/214 expression and the levels of both Ezh2 and Ki-67 proteins (56). The miR-199a/214 cluster has been shown to inhibit the production of Ezh2, β-catenin, or Ki-67, resulting in reduced proliferative activity of breast cancer cells (57, 58). In matched samples of breast cancer and adjacent normal tissues, it has been observed that the tumor suppressor genes GPER1 and miR-339 exhibit reduced expression in Luminal A/B and TNBC subtypes. Further mechanistic studies have uncovered that miR-339 enhances GPER1 expression in breast cancer cells by activating the GPER1 enhancer. This activation can be inhibited by excising the enhancer using the CRISPR/Cas9 technique (59). Multiple current studies confirm the tumor-suppressive effect of this cluster, highlighting its ability to impede the proliferation, migration, and invasion of breast cancer cells.

In a recent study of metastatic breast cancer, researchers analyzed plasma levels of miR-10b and miR-373. The investigation revealed a correlation between these miRNAs and the detection of lymph node metastasis, highlighting their potential as prognostic biomarkers (60). In addition, a study by Li et al. identified five different miRNAs (Let-7b-5p, miR-122-5p, miR-146b-5p, miR-210-3p, and miR-215-5p) in plasma samples from patients with breast cancer and discriminated them from normal controls (61). Furthermore, Bakr et al. investigated the diagnostic relevance of miR-373 in patients with breast cancer, going beyond its identification as a diagnostic biomarker to examine its interactions with the target genes vascular endothelial growth factor (VEGF) and cyclin D1. Their results indicated that miR-373 functions as an oncogene, thus confirming its significance as a biomarker for both breast cancer diagnosis and prognosis, particularly due to its targeting of VEGF and cyclin D1 (62). In addition, Zou et al. performed a comprehensive four-phase validation study in which 12 specific miRNA types were identified in serum samples from 216 patients with breast cancer and 214 normal controls. These miRNA types were identified using an exiqon miRNA quantitative PCR (qPCR) panel and subsequently validated by qRT-PCR. This particular panel of serum-detected miRNA molecules has significant potential for non-invasive breast cancer diagnosis (63).

## 3 Environmental chemicals and breast cancer

**Figure 2:**
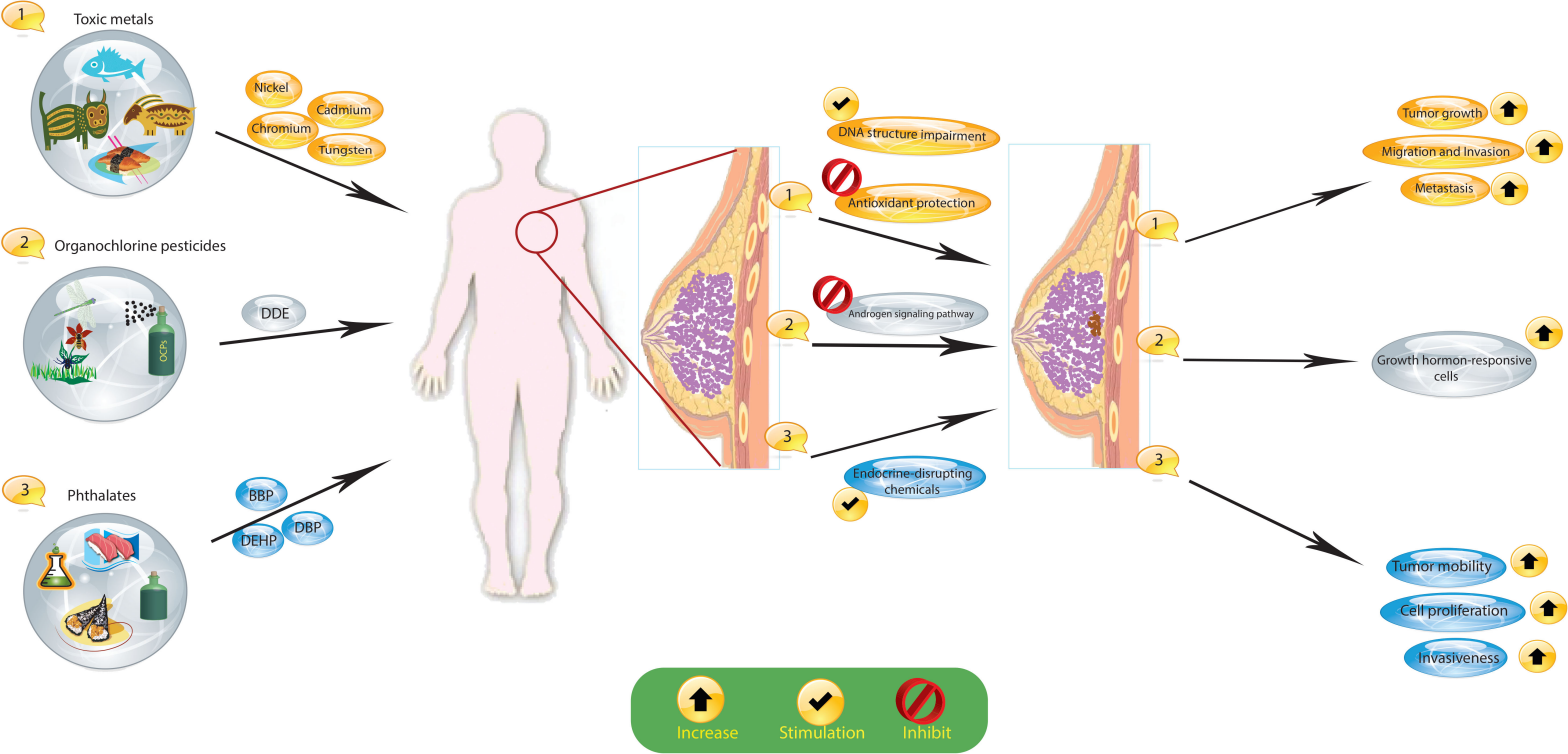
Exposure to certain environmental chemicals, such as organochlorine pesticides (OCPs), toxic metals, and phthalates, increases the risk of breast cancer. These substances cause hormonal changes and changes in the DNA of cells that lead to cancer. Dichlorodiphenyldichloroethane, DDE; dichlorodiphenyltrichloroethane, DDT; organochlorine pesticides, OCPs; butyl benzyl phthalate, BBP; di(n-butyl) phthalate, DBP; di(2-ethylhexyl) phthalate, DEHP.

The widespread prevalence of environmental chemical exposures is a major concern, with individuals encountering numerous established toxicants and several potentially risky chemicals with less thoroughly understood hazards throughout their lives. Growing epidemiologic data, combined with an enhanced comprehension of the mechanisms connecting toxicants to cancer development, indicate that exposure to specific environmental chemicals found in everyday products may increase the risk of developing breast cancer (**Figure 2**).

### 3.1 Organochlorine pesticides

An expanding body of epidemiologic research, coupled with a more sophisticated understanding of the links between carcinogenic substances and cancer growth, shows that exposure to certain environmental chemicals commonly found in everyday items may increase cancer susceptibility. Organochlorine pesticides (OCPs), which are synthetic organic compounds, belong to these chemicals. Globally, OCPs serve as insecticides, herbicides, termiticides, and fungicides in residential and agricultural settings. According to epidemiological studies, some OCPs, specifically dichlorodiphenyldichloroethane (DDE) and dichlorodiphenyltrichloroethane (DDT) may impact breast cancer development pathways, potentially affecting breast cancer prognosis and survival rates (64). Exposure to DDE has been associated with a heightened relative risk of lymph node involvement and more significant tumors, with the risk rising as the dosage increases (65). In addition, a recent study discovered a correlation between higher OCP levels in the blood and decreased overall survival rates (66). Current experimental evidence suggests that DDT can stimulate the growth of hormone-dependent breast cancer cells by disturbing the balance between estrogen and androgen, and by blocking the androgen signaling pathway that typically inhibits the growth in hormone-responsive cells (67).

### 3.2 Hexachlorobenzene

Hexachlorobenzene (HCB), a widely used organochlorine pesticide, was formerly utilized as a fungicide until the 1970s and continues to be discharged through various industrial processes. Studies have shown that exposure to HCB leads to cell proliferation and activates the insulin-like growth factor I (IGF-I) signaling pathway in MCF7 cells (68). Additionally, HCB activates the c-SRC/HER1/STAT5b and HER1/ERK1/2 signaling pathways in MDA-MB-231 cells, leading to cell migration in an AhR-dependent manner (69). The results obtained *in vitro* coincide with the observed activation of c-SRC, HER1, signal transducer and activator of transcription 5B (STAT5b), and extracellular signal-regulated kinase 1/2 (ERK1/2) signaling pathways, as well as the elevation of matrix metalloproteinase 2 (MMP2) and MMP9 protein levels caused by the pesticide.

### 3.3 Bisphenol A

Studies have shown a correlation between the exposure of breast cancer cells to bisphenol A (BPA) and a genetic expression pattern associated with aggressive tumors and unfavorable clinical results in patients with breast cancer (70). It is noteworthy that research conducted on MCF-7 cells, which have ER-positive attributes, has demonstrated that even minimal BPA exposure significantly increases cell proliferation (71). The heightened cell reproduction in MCF-7 breast cancer cells is attributed to the increase in genes involved in the cell cycle and the decrease in genes that have anti-proliferative effects (72, 73). It is notable that BPA has limited affinity for conventional estrogen receptors. Studies have indicated that, at ecologically significant levels, it can mitigate the adverse consequences of several chemotherapy drugs (like doxorubicin, cisplatin, and vinblastine) in ER-alpha-positive and -negative breast cancer cells. Critically, this outcome happens irrespective of conventional estrogen receptors and comprises the elevation of anti-apoptotic proteins (74). Studies have demonstrated that these cell types express alternative estrogen receptors, such as G-protein-coupled receptor 30 (GPR30) and members of the estrogen-related receptor family. However, the activity of GPR30, which is known to facilitate rapid estrogenic responses, remains unaffected in BPA-treated MDA-MB-231 and BT-549 cells. Furthermore, inhibiting GPR30 does not affect the BPA-induced expression of invasion-associated proteins. These findings suggest that GPR30 may not be involved in all BPA-triggered effects (75).

### 3.4 PhIP

The genotoxic compound known as 2-amino-1-methyl-6-phenylimidazo [4, 5-b] pyridine (PhIP), derived from cooked meat, has been associated with the development of colon, prostate, and mammary cancers in rat models. This carcinogenic effect results in profound biological changes such as increased proliferation, migration, invasion, and tumorigenicity with metastasis. Concurrently, there are molecular changes such as the upregulation of H-RAS gene expression, NOX-1, increased levels of reactive oxygen species (ROS), increased levels of HIF-1α and tumor necrosis factor-α, an increase in MMP-2 and MMP-9, and a decrease in E-cadherin expression (76). In another study, the application of PhIP to MCF-7 and T47D cells at sub-nanomolar concentrations resulted in a dose-dependent increase in invasion and migration potential (77).

### 3.5 Cigarette smoke

In developed countries, smoking is one of the major causes of death. In the United States, it is estimated that 30% of all cancer cases can be attributed to smoking (78). Cigarette smoke is a complex mixture of numerous chemicals that include toxic, potentially carcinogenic compounds such as polycyclic aromatic hydrocarbons (PAHs). During prolonged experimental exposure to cigarette smoke over several weeks, both benign (MCF10A and MCF12A) and malignant (MCF-7) mammary epithelial cells showed alterations, exhibiting mesenchymal features such as a “fibroblastoid” shape, increased unanchored growth, and increased motility and invasiveness (79). Analysis by flow cytometry revealed that as a result of cigarette smoke extract exposure, a CD44(hi)/CD24(low) population appeared in MCF10A cells, while a CD44+/CD49f+ population appeared in MCF7 cells (79). Cigarette smoke contains not only PAHs and other AHR ligands such as benzo[a]pyrene (BaP) but also nicotine, which is the primary addictive substance in this product. Studies have shown that nicotine promotes cancer cell proliferation and spreading in two human breast cancer cell lines (MCF7 and MDA-MB-468) by triggering a signaling pathway involving nAChR, SRC, and calcium. Recent research has shown that nicotine-treated MCF7 cells exhibit changes in cellular structure and motility, including F-actin rearrangement, and an increase in the MCF7 CD44 + CD24 cancer stem cell population (80).

### 3.6 2,3,7,8-tetrachlorodibenzo-p-dioxin

The chemical compound dioxin (TCDD) is recognized as one of the most potent carcinogens ever studied and holds the status of the most active congener among aryl hydrocarbon receptor (AHR) agonists. Epidemiologic study of patients with breast cancer found an association between TCDD concentrations in adipose tissue and the risk of metastasis, particularly in patients with a body mass index (BMI) equal to or exceeding 25 kg/m² (81). Notably, while TCDD exhibits potent tumor-promoting properties, it has been suggested to have a protective effect against breast cancer progression. In MCF7 cells, TCDD diminished the presence of the G-protein-coupled receptor CXCR4 and its distinctive chemokine ligand CXCL12, impeding the migration of cells in reaction to a CXCL12 gradient. Furthermore, TCDD reduced invasiveness, proliferation, and *in vitro* colony formation via the AHR pathway, irrespective of estrogen receptor status, while fostering the differentiation of a breast cancer cell line (82, 83). Furthermore, in a study investigating the effect of a single non-toxic dose of TCDD on the development of mammary tumors in female Sprague–Dawley rats treated with an oral dose of 7,12-dimethylbenzanthracene, it was observed that the mean tumor volume in rats with mammary tumors decreases under TCDD treatment compared to the control group (84). In contrast to the anti-tumor findings of TCDD, some studies showed that TCDD causes EMT, increased migration, mitochondrial dysfunction, and tumor dysfunction (85, 86). The divergent outcomes could be attributed to variances in the cell lines or assays employed and may also be influenced by various other factors, such as doses and treatment kinetics in cell culture.

### 3.7 Phthalates

Phthalates, such as butyl benzyl phthalate (BBP), di(n-butyl) phthalate (DBP), and di(2-ethylhexyl) phthalate (DEHP), are commonly used synthetic chemical contaminants as plasticizers and in food wraps and cosmetic formulations. These substances are believed to act as endocrine-disrupting chemicals and have been linked to heightened cell proliferation, increased tumor mobility, and the invasiveness of tumor cells (66, 87, 88). Moreover, phthalates are suspected to stimulate the proliferation and metastasis of breast cancer cells, promoting tumor progression by upregulating histone deacetylase 6 (HDAC6) (89). Moreover, the activation of peroxisome proliferator-activated receptors (PPARs) by these substances may play a role in the increased proliferation of MCF7 cells (90). Phthalates have demonstrated an ability to influence sensitivity to tamoxifen, potentially contributing to chemotherapy drug resistance by hindering tamoxifen-induced apoptosis in ER-positive MCF-7 cells (91).

### 3.8 Toxic metals

The presence of toxic metals such as zinc, lead, chromium, and nickel in breast cancer is associated with poor molecular prognostic factors (16). The carcinogenic effects of heavy metals are primarily manifested through mechanisms involving DNA structural impairment and the suppression of antioxidant protection. Moreover, there is evidence indicating that heavy metals may impact the expression of prognostically significant receptors in breast cancer tissue (92). Prior studies have shown that breast tissue, whether unaffected or affected by the tumor process, can accumulate heavy metals that affect DNA fragmentation levels and the survival of tumor cells (64). Moreover, the promotion of tumor growth by toxic metals has been linked to an increase in the expression of HER2/neu, p53, Ki-67, and O6-methylguanine-DNA methyltransferase and a decrease in ER-alpha and PR expression. Heavy metals have been discovered to advance the progression of breast cancer and diminish its sensitivity to treatment. Elevated concentrations of calcium, iron, copper, and zinc trace elements have been detected in neoplastic breast tissues (both malignant and benign) in comparison to normal tissues (17). Prolonged exposure to cadmium has been demonstrated to enhance the migration and invasion of breast cancer cells through the TGIF/MMP2 signaling axis (18). Moreover, tungsten has been noted for its impact on the tumor microenvironment, promoting increased metastasis in breast cancer. Specifically, in a breast cancer xenograft model, tungsten exhibited a modest delay in primary tumor growth. Despite not enhancing breast cancer cell proliferation or invasion *in vitro*, tungsten exhibited effects on tumor growth and metastasis *in vivo*. Lastly, a recent review on metal carcinogens suggested that metals could induce cancer stem cell (CSC)-like cells through dysregulated epigenetic mechanisms (93).

## 4 Interplay between MiRNA alterations and environmental chemicals

**Figure 3:**
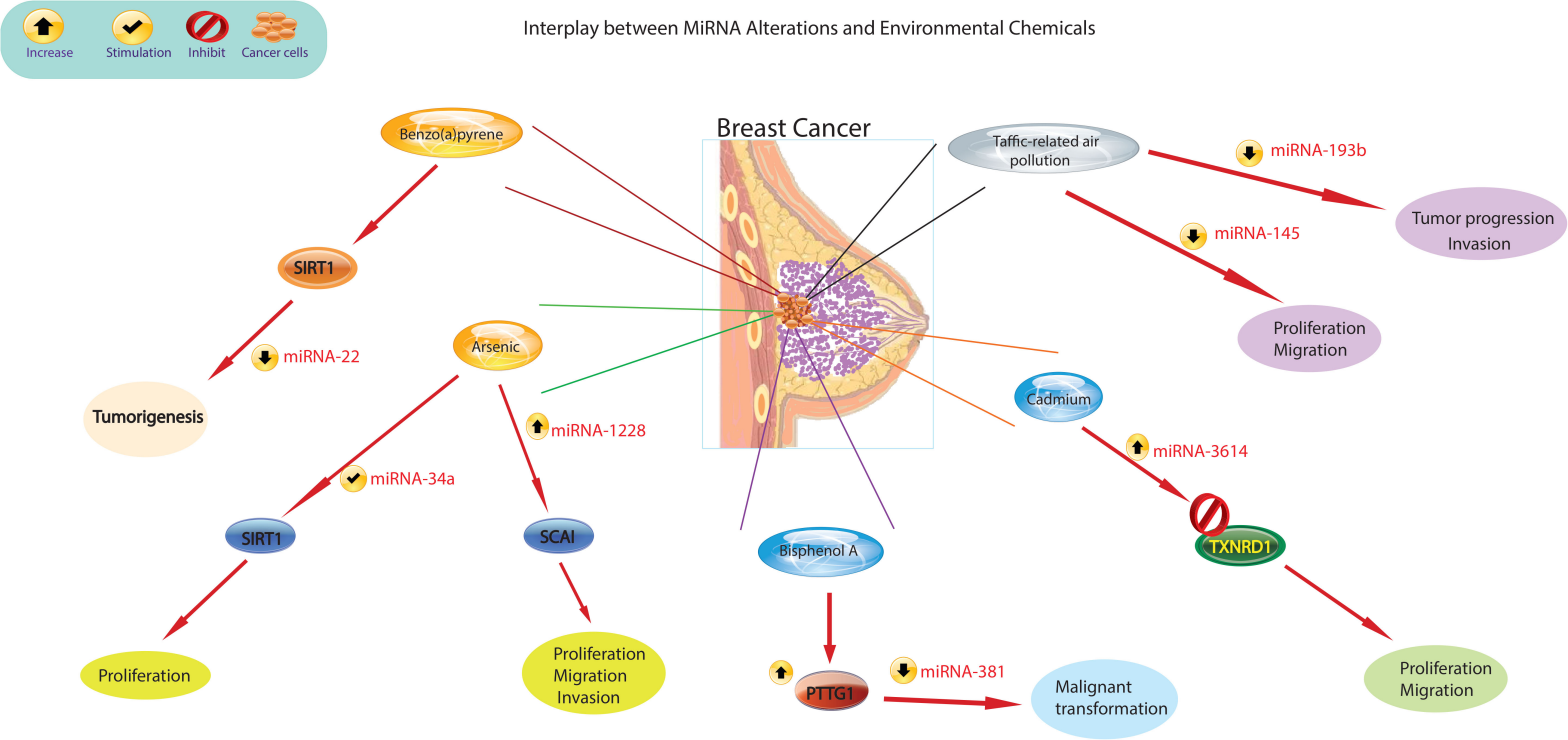
The figure illustrates different mechanisms by which environmental chemicals alter miRNA expression levels. Altered miRNA expression profiles could lead to aberrant gene expression patterns, disruption of cellular processes such as proliferation, differentiation, and apoptosis, and ultimately contribute to the development of breast cancer.

There is growing evidence that exposure to environmental chemicals poses significant risks to human health and is associated with a range of diseases. Epidemiologic and occupational health studies have specifically identified certain carcinogens, for example metals, organic pollutants, cigarette smoke, pesticides, and pharmaceuticals, as significant contributors to the development of cancer in humans. Recent epidemiological research has shed light on the role of chemical exposures in cancer development. For example, bladder cancer has been linked to exposure to arsenic (94), while BPA has been linked to breast cancer (95). Environmental chemicals can directly bind to miRNAs, altering their stability and functionality. This interaction can disrupt miRNA-mediated gene regulation and lead to dysregulated expression of target genes (**Figure 3**). A large body of research has consistently shown that miRNA expression is dysregulated in cancer and that these altered miRNAs play a critical role in cancer initiation and progression. These molecules can act as either oncogenes or tumor suppressors. Ongoing studies are rapidly expanding our knowledge of these altered miRNAs in various human cancers, including lung, liver, and breast cancer (96). For example, activation of miR-31 has been identified as a mechanism that promotes mammary stem cell expansion and breast tumor development by inhibiting Wnt signaling antagonists (97).

Recent scientific evidence has linked exposure to traffic-related air pollution (TRAP) to an increased risk of lung and breast cancer. It has been observed that those exposed to TRAP have reduced plasma levels of miR-145-5p and miR-193b-3p (98). The observed decrease in plasma levels of miR193b-3p in 85% of the subjects in relation to higher TRAP exposure levels is significant, as the downregulation of this miRNA in breast tissue is recognized to enhance tumor progression and invasion in human breast cancer (99). Similarly, the decreased plasma levels of miR-145-5p in 80% of participants are noteworthy, given that this miRNA serves to inhibit the growth and migration of breast cancer cells (100). Additionally, miR-145-5p has been found to inhibit the proliferation of non-small cell lung cancer cells by targeting the oncogene c-Myc, leading to the suggestion that increasing the expression of miR-145 could be a novel approach for the treatment of lung cancer (101). Furthermore, overexpression of miR-638 was found in peripheral lymphocytes of individuals exposed to PAHs, which enhances BaP-induced cellular DNA damage and may facilitate carcinogenesis by targeting breast cancer (102). Previous studies have identified miR-22 as an important regulator in inhibiting tumorigenesis and enhancing radiosensitivity of breast cancer cells by targeting sirtuin 1 (SIRT1) (103). Chronic exposure of cells to BaP has been shown to induce changes in the cell cycle, contributing to BaP-induced malignant transformation. Importantly, the regulation of the cell cycle by the p53/miR-34c axis has been implicated in the malignant transformation of cells induced by BaP. Furthermore, it has been demonstrated that either increased miR-34c or repression of cyclin D effectively inhibits BaP-induced malignant transformation. These findings underscore the critical role of the p53/miR-34c axis and cyclin D in mediating the effects of BaP on the cell cycle and malignant transformation, suggesting potential targets for intervention to mitigate the adverse effects of BaP exposure (104). Studies have shown that asbestos exposure may lead to respiratory cancers and mesothelioma of the lung. Alterations in the expression of certain miRNAs have been observed in the breast cancer MCF-7 and hepatoma HepG2 cell lines treated with nonylphenol (105). Studies carried out in the last decade have revealed that arsenic impacts the pattern of histone acetylation, methylation, and phosphorylation in exposed cells. Importantly, these changes have been linked to alterations in gene expression (106). These findings indicate that arsenic toxicity disrupts the regulatory function of chromatin remodeling complexes, particularly affecting the activity of histone-modifying proteins. Furthermore, in conjunction with changes in miRNA expression (107), these studies offer compelling evidence for a connection between arsenic-induced epigenetic dysregulation and the development of cancer. This revelation illuminates the potential role of arsenic-induced epigenetic alterations in carcinogenesis and emphasizes the importance of comprehending the mechanisms through which arsenic influences chromatin structure and gene regulation. Furthermore, studies by Herbert and collaborators (108) showed that arsenic exposure leads to changes in the epigenetic regulation of SIRT1 expression initially through structural chromatin reorganization at the miR-34a gene promoter. Over time, this reorganization was influenced by changes in methylation of the miR-34a and SIRT1 genes. Differential expression of miR-1228, miR-1254, and miR-645 was observed in human keratinocytes exposed to chronic low arsenic for 3 to 7 weeks. MiR-1228 has been implicated in breast cancer induction and regulation of SCAI mRNA and protein levels, miR-1254 has been associated with non-small cell lung cancer, and miR-645 has been described as a promoter of cell invasion and metastasis (109).

The reports present evidence indicating that elevated expression of pituitary tumor-transforming gene-1 (PTTG1) promotes breast cancer malignancy by augmenting the breast CSC population and inducing EMT through the activation of the PI3K/AKT pathway (110). In addition, previous studies have shown that PTTG1 is modulated by multiple miRNAs to promote cancer initiation and development. Recent research by Hu et al. showed that miR-329 directly targets PTTG1, leading to the inactivation of the MAPK pathway in cholangiocarcinoma (111). Similarly, miR-186 was shown to modulate PTTG1 protein expression and promote the migration and invasion of lung carcinoma cells (112). A study supported the role of global miRNA changes in response to BPA treatment in MCF-7 breast cancer cells (113), suggesting a potential contribution of the miRNA-PTTG1 pathway to BPA-mediated breast cancer development and progression.

A study by Deng et al (114). investigated the effect of BPA on breast cancer cell proliferation and found that BPA exposure significantly promoted MCF-7 cell proliferation and migration, but not invasion. The increased expression of PTTG1 upon BPA exposure was found to be caused by miR-381-3p inhibition. Furthermore, miR-381-3p expression was found to be low and inversely correlated with PTTG1 expression in breast cancer tissues. These results indicate that BPA promotes high PTTG1 expression and alters the cell cycle to promote MCF-7 cell proliferation by inhibiting miR-381-3p expression. This implies the potential role of miRNA-PTTG1 signaling pathway in BPA-mediated breast cancer development, shedding light on a novel mechanism by which BPA may contribute to breast cancer proliferation and progression.

In a recent study, Zhuo-Zhi Liang and colleagues revealed that cadmium triggered epigenetic alterations in breast cancer cells. Their investigation identified only two markedly decreased miRNAs in MCF-7 cells treated with 60 μM cadmium for 72 h, as determined through microarray analysis (115). Nonetheless, microarray findings from a study demonstrated that T-47D cells treated with 10 μM cadmium for 72 h exhibited downregulation in 29 miRNAs, while 93 miRNAs showed a significant increase (16). The differences observed in the miRNA expression profiles of MCF-7 and T-47D breast cancer cells following cadmium treatment could be attributed to multiple factors. First, the distinct responses observed in the two cell lines may be linked to inherent differences in their sensitivity to cadmium exposure. It is possible that the T-47D cell line exhibits greater susceptibility to cadmium-induced epigenetic modifications compared to MCF-7 cells. Additionally, the discrepancy in miRNA expression patterns could also be influenced by the variance in the cadmium concentrations used for treatment. The selection of a lower dose of cadmium in the T-47D cell line model, which may better replicate typical exposure levels in human breast tissue, could indeed contribute to the observed differences. These factors collectively emphasize the importance of considering cell line-specific responses and exposure levels in understanding the epigenetic impacts of cadmium in breast cancer cells. Taken together, environmental chemicals may induce epigenetic changes that lead to alterations in miRNA and subsequently contribute to the development of breast cancer (**Table 2**). Further research is needed to identify specific concentrations of environmental chemicals that promote carcinogenesis and to elucidate key molecular mechanisms for the effective treatment of breast cancer.

**Table 2:** Several studies have explored alterations in miRNA expression and their correlation with breast cancer development in the presence of environmental chemicals.

Study	Chemical carcinogens	Type of sample	Upregulated	Downregulated	miRNA function	Result	Ref.
Deng et al. (2021)	BPA	MCF-7 cells	**-**	miR-381-3p	Targets PTTG1 and CDC20 genes	BPA exposure significantly promoted MCF-7 cell proliferation and migration but not invasion.	(114)
Krauskopf et al. (2018)	Traffic-related air pollution	Human	**-**	miR-145-5p, miR-193b-3p	c-Myc	miRNAs as novel biomarkers for environmental health risk assessment.	(98)
Li et al. (2012a)	Arsenic	HUVECs	miR-638	**-**	Targets breast cancer, DNA repair	miRNA expression might play a crucial role in arsenic-induced vascular injury.	(102)
Zhang et al. (2017)	Benzo(a)pyrene	MDA-MB-231/MCF-7 cells	**-**	miR-22	Targets SIRT1	miR-22 suppresses tumorigenesis and improves breast cancer cells by targeting sirt1	(103)
Lv et al. (2017)	Cigarette smoke	MaSC/mammary tumors	miR-31	**-**	Targets Prlr/Stat5, TGFβ, Wnt/β-catenin signaling pathways, and the DKK1 gene	Mammary epithelial proliferation and MaSC expansion at the expense of differentiation *in vivo*.	(116)
Juhasz et al. (2012)	DMBA	CBA/CA H2(k) inbred mice	146a, let-7a	**-**	Ras, c-myc, CCR7/MAPK	miRNA expression can be regarded as an early effect of exposure to chemical carcinogens.	(117)
Tilghman et al. (2012)	BPA/DDT	MCF-7 cells	miR-149, miR-638, miR-663, miR-1915	let-7 g, let-7f, miR-21, miR-26b, miR-342-3p	Targets Wnt, p53, TGF-b, tumor suppressor	DDT and BPA alter the miRNA profiles in MCF-7 cells	(113)
Yue et al. (2022)	Cadmium	T-47D and MCF-7 cells	–	miR-374c-5p	miR-3614-5p inhibition caused an increase in TXNRD1 expression upon Cd exposure in T-47D and MCF-7 cell lines	TXNRD1 expression was suppressed by miR-3614-5p, and the inhibition of TXNRD1 significantly reduced the proliferation and metastatic potential of breast cancer cells following exposure to cadmium.	(118)

Reference (Ref); Bisphenol A (BPA); 7,12-dimethylbenz(α)anthracene (DMBA); human umbilical vein endothelial cells (HUVECs); dichlorodiphenyltrichloroethane (DDT).

## 5 Challenges and future directions

Investigating the impact of miRNA alterations and environmental factors on breast cancer is a multifaceted task in the current scientific landscape. A key obstacle arises from the intricate nature of miRNAs, tiny molecules that intricately regulate gene expression. The diverse functions they perform in cellular processes contribute to the complexity of understanding their involvement in cancer. Researchers face the challenge of elucidating how specific modifications in miRNAs influence the onset, progression, and response to treatment of breast cancer (119). The variability in patterns of miRNA expression among individuals further complicates the research endeavor. Tackling this complexity necessitates extensive studies encompassing large and diverse populations to yield meaningful findings. Furthermore, the influence of environmental factors on miRNA alterations and their association with breast cancer are gaining substantial attention. Factors such as pollutants, diet, and lifestyle choices have the potential to affect miRNA expression, thereby influencing cancer risk (120). However, identifying the precise environmental factors and unraveling the underlying mechanisms present significant obstacles. Conducting comprehensive, long-term investigations that monitor individuals’ environmental exposures and miRNA profiles is crucial; however, it requires substantial resources and time. Additionally, integrating extensive data from genomics, transcriptomics, and epigenomics to gain a comprehensive understanding of how miRNA alterations interact with environmental influences poses a formidable computational challenge. Analyzing these intricate datasets necessitates sophisticated bioinformatics tools and expertise. By overcoming these challenges, researchers will be able to unravel the intricate connections between miRNA alterations and environmental impacts. This breakthrough promises to open up new avenues for personalized approaches to breast cancer prevention and treatment.

## 6 Conclusion

The study of changes in miRNAs and their intricate links to breast cancer incidence and environmental factors provides valuable insights into the interplay between genetic components and environmental exposures. This updated evaluation highlights the vital role of miRNAs in regulating gene expression and how their dysfunction may contribute to the development and advancement of breast cancer. Identifying conclusive miRNA patterns associated with environmental exposure enhances our understanding of the disease and opens possibilities for early detection and personalized prevention and treatment strategies. By addressing the genetic and environmental factors that affect shifts in miRNA, there is potential to develop more effective prevention, early detection, and treatment strategies for individuals with breast cancer. This can ultimately lead to an improved prognosis and quality of life. Further investigation aimed at elucidating the relationships between changes in miRNA expression levels and environmental exposures will provide novel insights and establish a foundation for improved prevention, diagnosis, and treatment strategies for breast cancer.

## Author contributions

NA: Data curation, Investigation, Resources, Software, Writing – original draft. SS: Data curation, Investigation, Writing – original draft. BD: Data curation, Investigation, Resources, Software, Writing – original draft. VK: Data curation, Investigation, Visualization, Writing – review & editing. CA: Conceptualization, Data curation, Investigation, Writing – review & editing. BS: Conceptualization, Project administration, Supervision, Visualization, Writing – review & editing. AN: Conceptualization, Supervision, Visualization, Writing – review & editing.
